# Fucosylation Promotes Cytolytic Function and Accumulation of NK Cells in B Cell Lymphoma

**DOI:** 10.3389/fimmu.2022.904693

**Published:** 2022-06-15

**Authors:** Xing Tong, Yuhua Ru, Jianhong Fu, Ying Wang, Jinjin Zhu, Yiyang Ding, Fulian Lv, Menglu Yang, Xiya Wei, Chenchen Liu, Xin Liu, Lei Lei, Xiaojin Wu, Lingchuan Guo, Yang Xu, Jie Li, Peng Wu, Huanle Gong, Jia Chen, Depei Wu

**Affiliations:** ^1^ National Clinical Research Center for Hematologic Diseases, Jiangsu Institute of Hematology, The First Affiliated Hospital of Soochow University, Suzhou, China; ^2^ Institute of Blood and Marrow Transplantation, Collaborative Innovation Center of Hematology, Soochow University, Suzhou, China; ^3^ Key Laboratory of Stem Cells and Biomedical Materials of Jiangsu Province and Chinese Ministry of Science and Technology, Suzhou, China; ^4^ Department of Pathology, The First Affiliated Hospital of Soochow University, Suzhou, China; ^5^ Department of Molecular Medicine, The Scripps Research Institute, La Jolla, CA, United States

**Keywords:** fucosylation, NK cells, lymphoma, tumor immunotherapy, graft-versus-tumor effect

## Abstract

Natural killer (NK) cells have been demonstrated as a promising cellular therapy as they exert potent anti-tumor immune responses. However, applications of NK cells to tumor immunotherapy, especially in the treatment of advanced hematopoietic and solid malignancies, are still limited due to the compromised survival and short persistence of the transferred NK cells *in vivo*. Here, we observed that fucosyltransferase (FUT) 7 and 8 were highly expressed on NK cells, and the expression of CLA was positively correlated with the accumulation of NK cells in clinical B cell lymphoma development. *Via* enzyme-mediated *ex vivo* cell-surface fucosylation, the cytolytic effect of NK cells against B cell lymphoma was significantly augmented. Fucosylation also promoted NK cell accumulation in B cell lymphoma-targeted tissues by enhancing their binding to E-selectin. Moreover, fucosylation of NK cells also facilitated stronger T cell anti-tumor immune responses. These findings suggest that *ex vivo* fucosylation contributes to enhancing the effector functions of NK cells and may serve as a novel strategy for tumor immunotherapy.

## Introduction

Allogeneic hematopoietic stem cell transplantation (allo-HSCT) is one of the most curative therapies for malignant hematological diseases, predominantly in lymphoma and leukemia, for their potent graft-versus-tumor (GVT) effects. B cell acute lymphoblastic leukemia (B-ALL), an advanced B-cell malignancy, has a high relapse rate and poor prognosis after allo-HSCT (1). Subsequently, second transplantation provides long-term overall survival for 10%-40% of patients (2). Donor-derived T cells exhibit anti-tumor activities, whilst they can also induce graft-versus-host disease (GVHD), which has a significant impact on patients’ morbidity and mortality. New therapeutic strategies are needed to separate GVT and GVHD. Natural killer (NK) cells preserve critical GVT effects without aggravating GVHD (3). NK cells not only play a direct role in killing viruses and tumors but also act as a regulatory cell to mediate adaptive immune response by interacting with T cells, macrophages, dendritic cells (DCs), and endothelial cells (4). Although NK cell infusion has emerged as a promising immunotherapy for the treatment of hematologic malignancies, it is still limited due to their short lifespan and poor infiltration in solid tumors (5). The ability of immune cells to traffic to the tumor site usually relies on their robust binding to selectin, especially E-selectin (CD62E) expressed on activated endothelial cells (6). E-selection ligand, cutaneous lymphocyte antigen (CLA) must be sialofucosylated and presents the sialyl Lewis X (sLe^X^) epitope (7, 8). A straightforward glycan engineering approach based on exploiting α1,3-fucosyltransferase to transfer fucose residues from the guanosine diphosphate-fucose (GDP-fucose) donor onto the cell-surface α-2,3-sialyllactosamine acceptor substrate has been demonstrated effective and efficient to enforce sLe^X^ display in cell surface (9, 10). On the other hand, *ex vivo* cytokines pre-activation and genetically modification have been proven for promoting NK cell activity and infiltration (11, 12). Efforts are enforced to exploit the proliferation and migration of NK cells to generate potent anti-tumor properties.

Recent studies revealed that fucosylation play critical roles in the regulation of the development, functions, and trafficking of immune cells (13–15). For example, Diego et al. reported that *ex vivo* enforced sialofucosylation enhanced E-selectin binding of the modified CD19-CAR T cells, as well as their activity and homing to bone marrow (16). Likewise, Wu and coworkers also demonstrated that *ex vivo* fucosylation of NK-92MI cells to create the E-selectin ligand sLe^X^ on the cell surface promoted NK trafficking to bone marrow (17). However, the specific role and mechanism of fucosylation in modulating the NK cell-mediated anti-tumor immune response remains largely unexplored.

In this study, we discovered that NK cells were highly fucosylated and fucosylation was positively correlated with the infiltration of NK cells into the tumor microenvironment in B cell lymphomas. Fucosylation significantly promoted the cytolytic effect and the accumulation of NK cells. These findings suggest that *ex vivo* fucosylation contributes to enhanced effector functions of NK cells, which could be a novel strategy for tumor immunotherapy.

## Materials and Methods

### Animals

Female BALB/c (H2-Kd) and C57BL/6 CD45.2 (H2-Kb) mice were purchased from Shanghai Laboratory Animal Center (Shanghai, China). C57BL/6 CD45.1 (H2-Kb) mice were obtained from Beijing Vital River Laboratory Animal Technology Co. Ltd (Beijing, China). All mice used were aged 6-8 weeks and housed in a specific-pathogen-free environment and received acidified autoclaved water at Animal Facilities of Soochow University. All animal experiments were performed in accordance with the guidelines and approved by the Animal Care and Use Committee of Soochow University.

### Cell Line

A20 cells line (BALB/c B cell lymphoma, H2-Kd) was purchased from American Type Culture Collection (Manassas, VA, USA). Luciferase-expressing A20 cells were generated by lentiviral system and sorted by flow cytometry (BD FACS Aria III, BD Bioscience, San Jose, CA, USA). Both of these cells were cultured with RPMI 1640 medium with 10% FBS at 37°C in a 5% CO_2_ incubator.

### GVT Model

Murine GVT model was established as previously described (18). Briefly, BALB/c recipients received lethal irradiation of 650cGy (X-Ray, 325cGy per dose with 4h interval) and were injected intravenously with 5×10^6 bone marrow (BM) cells from C57BL/6 mice together with 1×10^6 A20 lymphoma cells or 5×10^6 A20-*luc^+^/yfp* cells, respectively. Survival of recipients were monitored daily.

### Fucosylation and Generation of NK Cells

NK cells were generated from bone marrow of CD45.2 C57BL/6 mice or CD45.1 C57BL/6 mice as described previously (19). T cells in BM components were depleted by EasySep™ Mouse CD90.2 Positive Selection Kit (STEMCELL Technologies, Vancouver, BC, Canada). T cell depleted (TCD)-BM cells were incubated in fucosylation solution (40ug/ml α-1,3-fucosyltransferase and 100 μM GDP-fucose in phosphate-buffered saline with 0.5% FBS) for 30 min at room temperature and then cultured for 7 days in the presence of IL-2 and Indomethacin. The success of cell-surface fucosylation was characterized by the increased expression of sLe^X^ residues, as assessed by flow cytometry, using CLA, recognized by the monoclonal antibody HECA-452 (20). Purity of CD3^−^ NK1.1^+^ NK cells was >95%. NK cells were washed three times with PBS before transfer.

### CFSE Labeling and *In Vivo* Proliferation Analysis

Donor NK cells were labeled with 5 mM CellTrace™ Violet Cell Proliferation kit (Invitrogen, Waltham, MA, USA) as described previously (19) and then transferred into tumor-bearing recipients. Four days post adoptive transfer, the proliferation of donor NK cells was analyzed by individual CTV generations.

### Cytotoxicity Assay

Control or fucosylated NK cells and A20-*luc^+^/yfp* cells were co-cultured in 96-well plates at different effector/target (E: T) ratios. After co-cultured for 6h, apoptosis of *yfp^+^* lymphoma cells were detected by Annexin V/PI Apoptosis Detection Kit (Vazyme, China). The cytotoxic activities of NK cells were represented by the apoptotic rates.

### Flow Cytometric Analysis

Single cell suspensions from the spleen and liver were acquired according to the methods previously described (21) and analyzed using flow cytometry. Antibodies used for flow cytometry staining including Percp-Cy5.5-anti-mouse-CD45.1, APC-Cy7-anti-mouse-CD11b, Percp-Cy5.5-anti-mouse-NK1.1, BV650-anti-mouse-H2-Kb were purchased from BD Bioscience (SanDiego, CA, USA); purified anti-mouse-CD16/32, APC-anti-mouseCD43, PE-anti-mouse-NKp46, APC-anti-mouse-CD107, FITC-anti-mouse-NKG2D, PE-anti-mouse-IFN-γ, PE/Cy7-anti-mouse-TNF-α, FITC-anti-mouse-CD62E, PE-anti-mouse-CD4, PE/Cy7-anti-mouse-CD44, APC/Cy7-anti-mouse-CD62L, Pacific Blue-anti-mouse-CD8a, FITC-anti-mouse-CD69 were purchased from Biolegend (San Diego, CA, USA); PE-Cy7-anti-mouse-Granzyme B, APC-anti-mouse-Perforin were purchased from eBioscience (San Diego, CA, USA). Recombinant Mouse E-Selectin, P-Selectin, and L-Selectin chimera were purchased from Biolegend (San Diego, CA, USA) for detecting the binding abilities. Samples were detected on a NovoCyte Flow Cytometer (ACEA Biosciences, San Diego, CA, USA) and data were analyzed by using Flowjo software (Flowjo, Ashland, OR, USA).

### Single Cell RNA Sequencing Analysis

Single cell RNA sequencing (scRNA-seq) data were available in a previous study that deposited in the GEO database (NCBI) repository, accession number GSE182434 and normalized by R package “Seurat”. After filtering cells with low numbers of total UMI counts, detected genes and low proportion of mitochondrial gene counts per cell, poor-quality cells were removed. Differential gene expression (DEG) testing was performed using the “FindMarkers” function in Seurat with a Wilcoxon test, and *p-*values were adjusted using Bonferroni correction. DEGs were filtered using a maximum adjusted *p*-value of 0.05. Enrichment analysis for the functions of the DEGs was conducted using the clusterProfiler (v3.12.0) R package and GSEApy (0.10.2) in Python3. Metascape (http://metascape.org/gp/index.html#/main/step1) was used to create gene ontology and cell type enrichment barplots using DGE of relevant groups. The gene sets were based on Gene Ontology terms in MSigDB, and all the gene sets with NESscores higher than 1 and a *p*-value less than 0.05 were included. scRNA-seq plots were generated using ggplot2 (v3.3.5). Cell interactions were performed by Cellphone DB.

### Morphology and Immunohistochemistry

The tissue wax obtained from the Department of Pathology, The First Affiliated Hospital of Soochow University. Formalin-fixed, paraffin-embedded (FFPE) tissues was used for morphological examination *via* hematoxylin-eosin (H&E) staining. The following antibodies were applied on BenchMark XT automated immunostainer (Ventana Medical Systems, Tucson, AZ, USA) with Cell Conditioning 1 heat retrieval solution (Ventana Medical Systems, Tucson, AZ, USA): CD56 antibody (clone number: 123C3, Gene Tech, China; Ready-to-use), CD8 antibody (clone number: SP16, Gene Tech, China; Ready-to-use) and CLA (clone number: HECA-452, BioLegend, USA). IHC results of CD8, CD56 and CLA were calculated as IHC score by multiplying the percentage of positive cells (0 to 100, recorded in the increment by 5%) with mean intensity (0, no staining; 1, weak staining; 2, moderate staining; 3, strong staining), and given a range from 0 to 300. Two pathologists (Xing Tong and Lingchuan Guo) were independently responsible for evaluating the morphological and immunohistochemistry (IHC) results. The use of human samples was approved by the Ethical Committee of The First Affiliated Hospital of Soochow University and all patients provided signed informed consents.

### Statistical Analysis

Data were analyzed using GraphPad Prism 9 software for Mac (GraphPad Software, San Diego, CA, USA). Unpaired Student’s *t*-test was used to investigate statistical significance. The Kaplan-Meier curve was used to analyze the survival of allo-HSCT. *p* < 0.05 was considered statistically significant (*), less than 0.01 or 0.001 was shown as ** or ***, respectively. Data are presented as means ± SD.

## Results

### Fucosylation Promoted Effector Functions of NK Cells in B Cell Lymphoma

**Figure 1:**
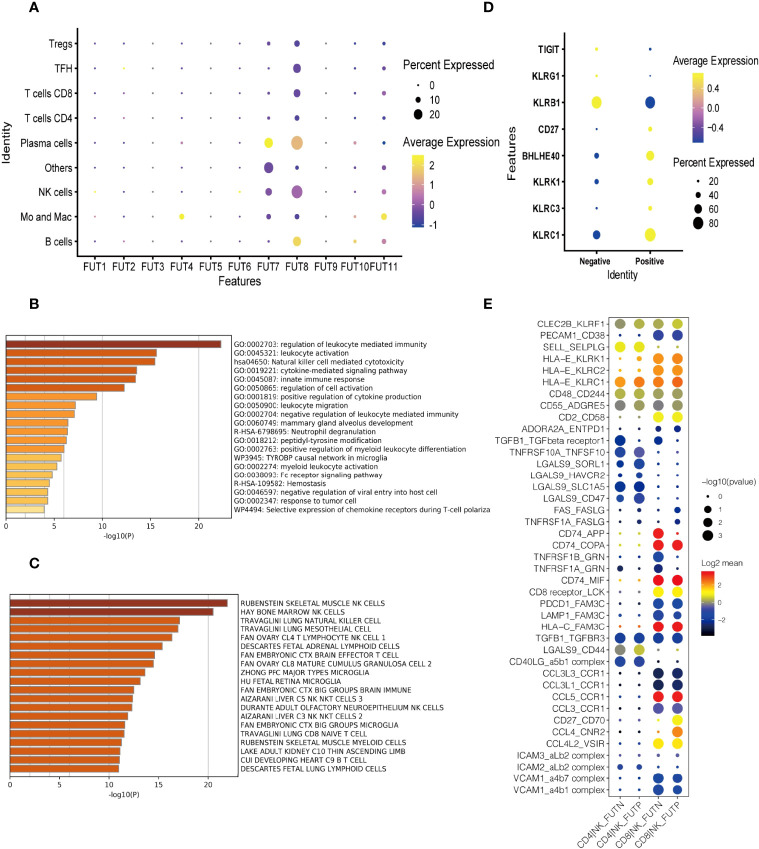
Fucosylation promoted effector functions of NK cells in B cell lymphoma. **(A)** Heatmap of relative expressions of different fucosyltransferases in each cluster defined by scRNA-seq analysis. The dot size represented the relative percentage of expressions. The color scale represented the expression level. **(B)** GSEA of the upregulated gene set in fucosylated NK cells versus normal NK cells. **(C)** Enrichment of cell type signatures in fucosylated NK cells versus normal NK cells. **(D)** Heatmap of relative expressions of NK effector genes. The dot size represented the relative percentage of expressions. The color scale represented the expression level. **(E)** The GO annotation of ligand-receptor pairs between NK cells and T cells. The dot size represented the adjusted *p*-value. The color scale represented number of genes.

To explore the role of immune cell fucosylation in B cell lymphomas pathophysiology, we first analyzed the expressions of individual fucosyltransferases in publicly available scRNA-seq database of 14 patients with diffuse large B cell lymphoma (DLBCL) and follicular lymphoma (FL) (22). We found that fucosyltransferases, including *FUT1, 2, 3, 5, 6, 9*, and *10* had extremely low expression levels on different immune cell subsets. *FUT4* was restrictedly expressed on monocytes and macrophages. Notably, *FUT7* was predominantly expressed on NK cells and plasma cells, which was responsible for α1,3 fucosylation to produce sLe^X^. Similarly, we observed *FUT8*, a specific fucosyltransferase for α-1,6 core fucose, was highly expressed on NK cells (**Figure 1A**). These results indicated that fucosylation may have a potential function in anti-tumor immune responses regulated by NK cells during the pathogenesis of B cell lymphoma. We next performed gene-set enrichment analysis (GSEA) with KEGG (Kyoto Encyclopedia of Genes and Genomes) pathway analysis to investigate the functions of fucosylated NK cells. Interestingly, the expression patterns of genes known to be involved in ‘nature killer cell mediated cytotoxicity’ and ‘regulation of leukocyte mediated immunity’ were increased with fucosylation of NK cells (**Figure 1B**). Cell type analysis further enriched NK cell signatures (**Figure 1C**). Moreover, we also observed an elevated expression of NK effector genes including *KLRC1*, *KLRC3*, *KLRK1*, *BHLHE40*, *CD27* and down regulation of inhibitory molecules including *KLRB*, *KLRG1*, and *TIGIT* (**Figure 1D**). Interactions between different cell subsets played critical roles in anti-tumor responses. Given that NK cells and CD8^+^ T cells are fundamental in anti-tumor immunity, we then performed cell-cell interaction analysis and calculated the numbers of receptor-ligand parings based on Cellphone DB to elucidate the redistribution of each kind of ligand-receptor interactions of fucosylated NK cells and T cells in B cell lymphoma (23). We observed interactions of cytokine receptors, immune checkpoints, and adhesion molecules between fucosylated NK cells and CD8^+^ T cells in B cell lymphoma (**Figure 1E**). Thus, these results suggest that fucosylation promotes NK cell effector functions and may have a potential regulatory role in facilitating T cell anti-tumor responses.

### Fucosylation Was Relevant to Infiltration of NK Cells in DLBCL

**Figure 2:**
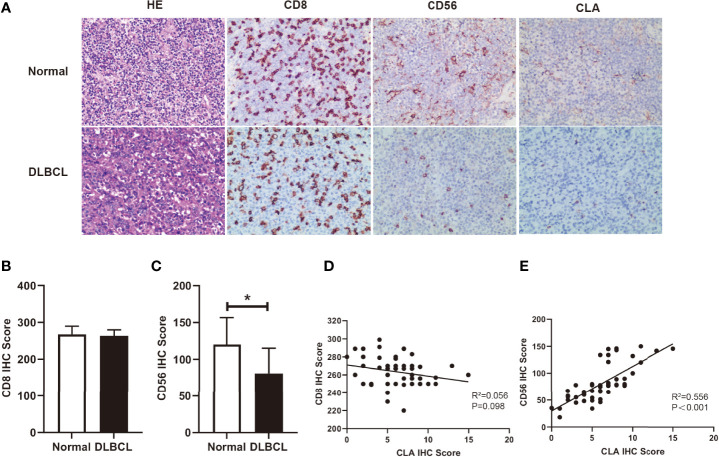
Fucosylation was relevant to infiltration of NK cells in DLBCL. **(A)** Representative images of morphology with H&E staining and immunohistochemical staining of CD8, CD56, and CLA between normal and DLBCL cases (magnification×200). **(B, C)** Statistics were based on the expression of CD8^+^ T cells and CD56^+^ NK cells in DLBCL(n = 50) and normal lymph node (n = 4) biopsies per field of each group. **(D)** Correlations of CLA positive cells and CD8^+^ T cells in DLBCL (*p *= 0.098, r^2 =^ 0.056, n = 50) **(E)** Correlations of CLA positive cells and CD56^+^ NK cells in DLBCL (*p *< 0.001, r^2 =^ 0.56, n = 50). Data are presented as mean ± SD. *p < 0.05.

It is reported that CLA is mainly expressed on memory/effector T cells and NK cells (24). With H&E staining, we observed that tumor cells were numerous on moderate to large lymphocytes proliferating diffusely, featured with marked heteromorphy, and large and deeply stained nuclei which distinguished from those small lymphocytes in backgrounds in DLBCL (**Figure 2A**). To further investigate the role of fucosylation in B cell lymphoma, we performed immunohistochemical staining to detect the correlations between fucosylation and CD8 or CD56. Our results showed that compared to normal control, CD8 expression in DLBCL remained strong and diffuse positive (**Figure 2B**), while CD56 expression decreased significantly amidst reactive backgrounds (**Figure 2C**). Moreover, weak and scattered expression of CLA was observed only on small lymphocytes in normal control, and further decreased in DLBCL (**Figure 2A**), which was in accordance with CD56 expression (**Figure 2E**). By contrast, there existed no correlations between the expression of CD8 and CLA (**Figure 2D**). These results implied the role of NK cells in DLBCL, which was potentially related to fucosylation.

### *Ex Vivo* Fucosylation Augmented Cytotoxic Activities of NK Cells

**Figure 3:**
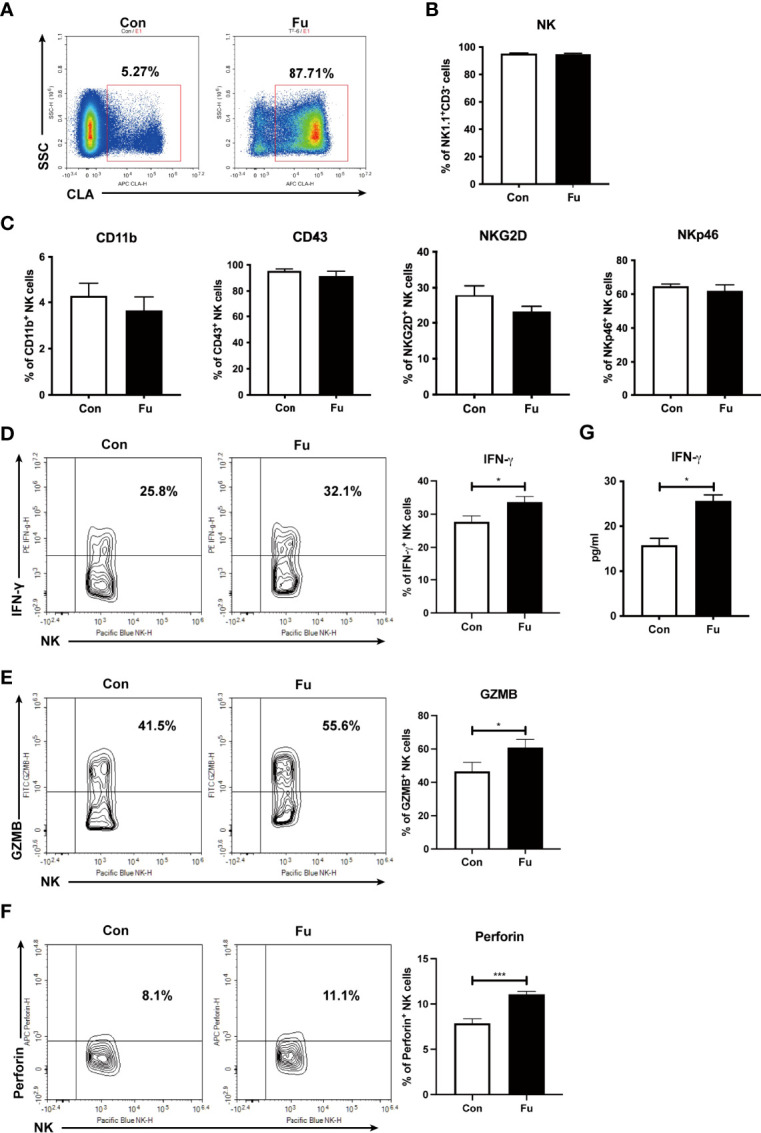
*Ex vivo* fucosylation augmented cytotoxic activities of NK cells. **(A)** Representative flow cytometry plots of control and fucosylated BM progenitor cells. **(B)** The percentage of NK1.1^+^CD3^-^ cells in the generated control and fucosylated NK cells. **(C)** Expressions of CD11b, CD43, NKG2D, NKp46 in control and fucosylated NK cells are shown. **(D, F**, **G)** Representative flow cytometry plots and statistics of the proportions of IFN-γ, GZMB and perforin productions in control and fucosylated NK cells are shown, respectively. **(E)** IFN-γ production in the superannuants of control and fucosylated NK cells were detected by ELISA (n = 4 per group). Data are representative of three independent experiments and shown as mean ± SD. *p < 0.05; ***p < 0.001.

Fucosylation is regulated by fucosyltransferases and GDP-fucose as the substrate (25). In order to investigate the function of fucosylation of NK cells, we incubated TCD-BM progenitors with H. pylori α-1,3-fucosyltransferases (26) and GDP-fucose for 30 min at room temperature. BM progenitors only expressed a relatively low level of fucosylation. *Ex vivo* fucosylation significantly promoted the CLA expression on cell surface as increased from 5.27% to 87.71% (**Figure 3A**). The purity of generated NK cells was high (more than 95%) (**Figure 3B**). There was no significant difference of CD11b and CD43 expression between the fucosylated and untreated groups, suggesting that fucosylation had no impact on the maturation of NK cells. Likewise, the activation markers NKG2D and NKp46 were not substantially altered by fucosylation (**Figure 3C**). However, production of IFN-γ, granzyme B as well as perforin were markedly increased in fucosylated NK cells as compared to those of the untreated controls (**Figures 3D-F**). We also observed an elevated IFN-γ secretion in the supernatant of fucosylated NK cells (**Figure 3G**). Collectively, these results demonstrated that *ex vivo* fucosylation promoted the cytotoxic abilities of NK cells.

### Fucosylated Donor NK Cells Enhanced GVT Effect Against B Cell Lymphoma

**Figure 4:**
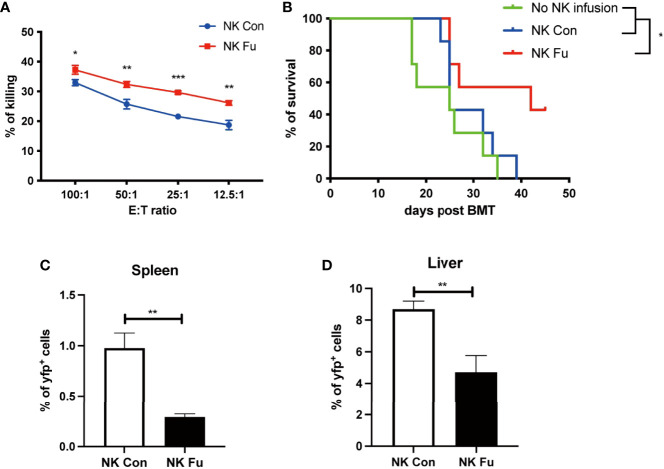
Fucosylated donor NK cells have enhanced GVT effect against B cell lymphoma. **(A)** Fucosylated NK cells and control NK cells were cocultured with A20 lymphoma cells at different ratios for 6 hours, respectively. Cytotoxicity of NK cells were evaluated by the apoptosis of yfp^+^ lymphoma cells as detected by flow cytometry. **(B)** BALB/c recipients were lethally irradiated and transplanted of 5x10^6 BM cells together with 5×10^6 A20-*luc^+/^yfp* cells. Recipients were transferred with or without 1x10^6 control NK cells or fucosylaed NK cells, respectively. Survival of recipients were monitored (n = 7-9 mice per group). **(C, D)** Proportions of *yfp* positive lymphoma cells in the spleen and liver were detected by flow cytometry 7 days post transplantation. Data are representative of three independent experiments and presented as mean ± SD. *p < 0.05; **p < 0.01; ***p < 0.001.

To evaluate the potential GVT effect of fucosylation on NK cells, we performed cytotoxicity assay to detect the direct killing abilities of NK cells against B cell lymphoma cells (A20 cells). We found that fucosylation significantly promoted the cytotoxic activities of NK cells at different E: T ratios compared to control NK cells (**Figure 4A**). Results of murine GVT model showed that compared to recipients without NK cells infusion, recipients receiving control NK cells have a trend of prolonged survival, but without statistical significance. By contrast, recipients that received the transfer of fucosylated NK cells exhibited prolonged survival and profound therapeutic effects compared to recipients receiving control NK cells (**Figure 4B**). The proportions of A20 cells were significantly decreased both in the spleen and liver in mice infused fucosylated NK cells compared with those in controls (**Figures 4C, D**). Thus, our results demonstrated that fucosylation of NK cells displayed enhanced anti-tumor responses both *in vitro* and *in vivo*.

### Fucosylation Promoted Donor NK Cells Accumulation After Allo-HSCT

**Figure 5:**
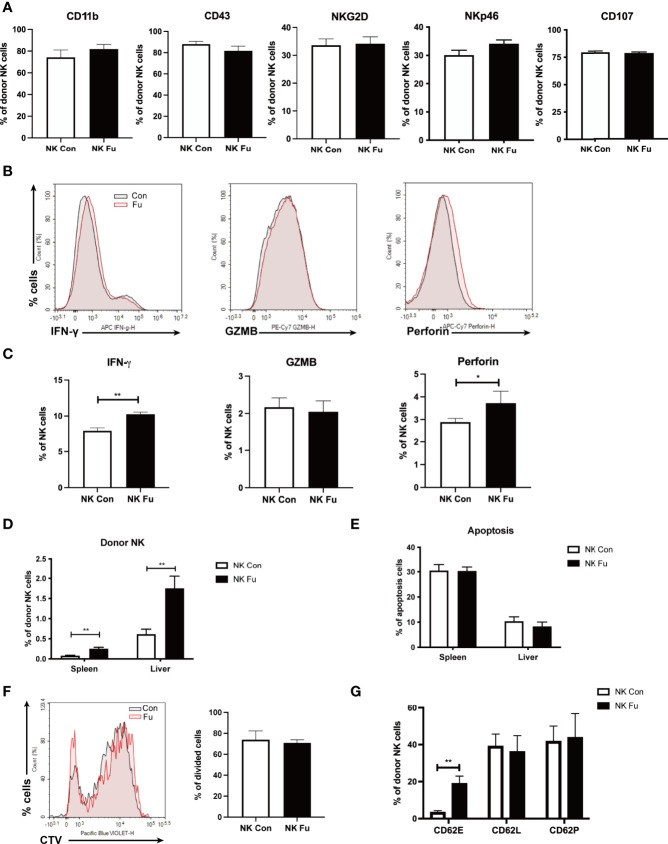
Fucosylation promoted donor NK cells accumulation after allo-HSCT. BALB/c recipients were lethally irradiated and transplanted of 5x10^6 BM cells together with 5×10^6 A20-*luc^+^/yfp* cells. Recipients were transferred with 1x10^6 control NK cells or fucosylaed NK cells, respectively. **(A)** Expressions of CD11b, CD43, NKG2D, NKp46, and CD107 of NK cells in the spleen from mice infused with control or fucosylated NK cells (n = 5-6 per group). **(B, C)** Representative flow cytometry plots and summary data of the frequencies of IFN-γ, GZMB and perforin of NK cells from mice infused with control or fucosylated NK cells (n = 5-6 per group). **(D)** Percentages of donor NK cells in the spleen and liver in recipients were detected by flow cytometry 7 days after allo-HSCT (n = 5-6 per group). **(E)** Percentages of apoptosis of control and fucosylated NK cells in spleen and liver are depicted. **(F)** Control and fucosylated NK cells were labeled with CTV. The proliferation of CTV labeled control and fucosylated NK cells were detected by flow cytometry 4 days post transfer (n = 4-6 per group). **(G)** Binding abilities of control and fucosylated NK cells to CD62E, CD62L and CD62P *in vivo* was detected by flow cytometry (n = 3-6 per group). Data are representative of three independent experiments and shown as mean ± SD. *p < 0.05; **p< 0.01.

To investigate the mechanisms of promoting GVT effect with *ex vivo* fucosylation of NK cells, we assessed the properties of NK cells 7 days post transplantation. We found that neither the expressions of maturation markers (CD11b and CD43) nor the expressions of activation markers (NKG2D and NKp46) differed between the fucosylated and the control NK cells isolated from the recipient mice (**Figure 5A**). In addition, we also found similar levels of CD107a expressed on NK cells between these two groups. Consistent with our *in vitro* results, the production of IFN-γ and perforin was elevated in the fucosylated NK cells (**Figures 5B, C**). Significantly, we found the frequency of NK cells in the spleen and the liver was substantially increased in the recipient mice that received fucosylated NK cells when compared to those receiving control NK cells (**Figure 5D**). To determine the origin of NK cells increased in these organs, we then labeled donor NK cells with CTV before transfer. The fucosylated NK cells exhibited a similar rate of apoptosis and proliferation to that of the control NK cells (**Figures 5E, F**). However, the fucosylated NK cells had increased affinity to E-selectin rather than P-selectin or L-selectin, which hinted at superior accumulation ability of the fucosylated NK cells compared to the controls (**Figure 5G**). These results suggested that, instead of enhanced proliferation or hindered apoptosis, fucosylation promoted the accumulation of NK cells in lymphoma-involved organs by enhanced binding to E-selectin *in vivo*.

### Fucosylation of NK Cells Triggered T Cell Anti-Tumor Immune Responses

**Figure 6:**
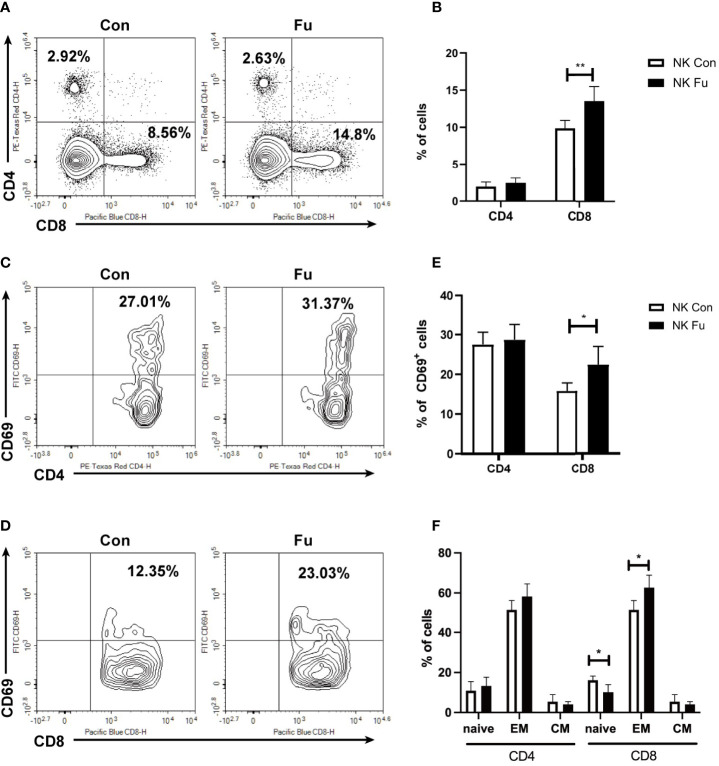
Fucosylation of NK cells triggered T cell anti-tumor T cell responses. BALB/c recipients were lethally irradiated and transplanted of 5x10^6 BM cells together with 5×10^6 A20-*luc^+^/yfp* cells. Recipients were transferred with 1x10^6 control NK cells or fucosylaed NK cells, respectively. **(A, B)** Representative flow cytometry plots and summary data of the proportions of CD4^+^ and CD8^+^ T cells in recipients 7 days post allo-HCST were shown (n = 5-7 per group). **(C–E)** Representative figures and summary data of the proportions of CD69^+^CD4^+^ T cells and CD69^+^CD8^+^ T cells are depicted (n = 5-7 per group). **(F)** Populations of naïve, effector memory and central memory CD4^+^ and CD8^+^ T cells were detected by flow cytometry in recipients received control and fucosylated NK cells 7 days post allo-HCST (n = 5-7 per group). Data are representative of three independent experiments and shown as mean ± SD. *p < 0.05; **p < 0.01.

Results of scRNA-seq indicated that engagements of fucosylated NK cells and T cells might facilitate the anti-tumor immunity. Hereby, we assessed the early T cell response 7 days post adoptive transfer. We found that the percentage of CD8^+^ T cells was significantly increased in recipients with fucosylated NK cells, whereas the population of CD4^+^ T cells was not altered (**Figures 6A, B**). Compared to mice with control NK cells, CD8^+^ T cells of mice with fucosylated NK cells also exhibited a more activated phenotype with elevated expression of CD69 (**Figures 6C–E**). Accordingly, we observed a decrease of naïve CD8^+^ T cells and an increase of effector memory CD8^+^ T cells in recipient mice that received fucosylated NK cells compared with those receiving control NK cells (**Figure 6F**). However, similar alterations were not observed in CD4^+^ T cells. Thus, fucosylation of NK cells probably contributed to the activation of CD8^+^ T cells, which further promoted the anti-tumor immune response.

## Discussion

NK cells were defined as a distinct lymphocyte subset in 1975, which are capable of directly recognizing and killing tumor cells without previous sensitization (27). It has been demonstrated that NK cells play critical roles in GVT responses without aggravating GVHD (3, 28). NK cells can directly eliminate tumor cells by cytotoxic effect through releasing perforin and granzymes. In addition, by producing IFN-γ, NK cells also facilitate the activation and differentiation of cytotoxic T lymphocytes to restrict tumor growth (29). Apart from these direct manners, NK cells can assist in indirect regulation of T cell responses, such as interactions with B cells, triggering DC maturation and antigen cross presentation (30). Indeed, we observed an increased activation of CD8^+^ T cells in recipient mice infused with fucosylated NK cells as compared with those infused with the control, untreated NK cells. However, clinical applications of NK cells infusion in tumor immunotherapy, especially in the treatment of advanced hematopoietic and solid malignancies, are still challenging due to their limited ability for survival or accumulation. It is supposed that *ex vivo* modification may provide potentials to ameliorate their ability and enhance their anti-tumor properties.

Fucose is a natural 6-deoxy hexose, characterized by the form of L-configuration and lacks a hydroxyl group on the carbon at the 6-position (C-6). There exists a variety of fucose types in mammal tissues, including skin and nervous system. Fucose can be incorporated into the terminal portions of N-, O-, or lipid-linked oligosaccharide chains as a terminal modification of glycan structures (31). Besides, levels of L-fucose in serum and urine can be a valuable biomarker of alcoholic liver disease, hepatocarcinoma, cirrhosis, and gastric ulcers (32, 33). Fucosylation is an enzymatic process catalyzed by fucosyltransferase (FUTs). FUT family, including FUT1 to FUT11, are fucosylation synthases which are responsible for forming glycosidic linkages between saccharides and other saccharides, peptides, and lipids (34). They are involved in proliferating cancer cells and play an important role in tumor metastasis (35, 36). It was reported that expression of FUT3/6/7 was a poor prognostic indicator, but higher FUT4 expression was a favorable prognostic factor in AML patients who received chemotherapy alone (37). FUT7 promoted the proliferation, migration, invasion, and EMT of bladder cancer cells, and positively correlated with immune cell infiltration levels (CD8^+^ T cells, CD4^+^T cells, macrophage, neutrophil, and DCs) (38). In our study, we found that FUT genes wereassociated with immune infiltration in B cell lymphoma. FUT4 was restrictedly expressed on monocytes and macrophages, while FUT7 and FUT8 were predominantly expressed on NK cells through scRNA-seq analysis. Interestingly, we found that there was scarcely any expression of CLA on the lymphoma cells by immunohistochemical staining.

Fucosylation is involved in the formation of ABO blood group H antigen, Lewis blood group antigen, selectin-mediated leukocyte extravasation or homing, host pathogen interaction, and signal pathway modification (39). *Ex vivo* fucosylation has emerged as an enabling way to facilitate the trafficking and tumor infiltration of adoptively transferred immune cells. In the pioneering work of Xia, Shpall, and coworkers, cord blood hematopoietic cells fucosylation mediated fucosyltransferase-VI effectively accelerated neutrophil and platelet engraftment after transplantation both in animal models and clinic trials. Likewise, Sackstein applied similar strategies to create sLe^X^ on human multipotent mesenchymal stromal cells to enhance their trafficking and bone marrow engraftment. In the scenarios of adoptive T cell transfer, *ex vivo* fucosylation was effective to enhance anti-GVHD potency of human regulatory T cells (13, 14, 40, 41). It also reported that fucosylated CD19-CAR T-cell acquired improved activity and prolonged persistence *in vivo* (16). However, little is known about fucosylation in NK cell immunity.

In the current study, we discovered that in clinical B cell lymphoma FUT7 responsible for creating sLeX was highly expressed on NK cells and CLA that presented sLeX was positively correlated with the accumulation of NK cells in tumor bed. By employing recombinant H. pylori α1,3-fucosyltransferase for *ex vivo* fucosylation, we successfully introduced sLeX onto murine NK cells. *Ex vivo* fucosylation significantly enhanced the cytolytic activity of the treated NK cells as well as their infiltration into lymphoma-involved organs such as the spleen and the liver. Moreover, fucosylated NK cells exhibited superior activities to restrict tumor growth by facilitating CD8^+^ T cell activation. However, we observed that fucosylation on the cell surface persisted less than 2 weeks in our study (data not shown). How to prolong the persistence of fucosylation needs to be explored in further studies. Besides, the molecular mechanism through which cell-surface fucosylation enhances NK effector function remains to be explored. There is a possibility that fucosylation enforces stronger interactions between NK cells and target cells, which results in the formation of a stronger immunological synapse and thereby better tumor cell killing. Because H. pylori α1,3-fucosyltransferase possesses unprecedented donor substrate promiscuity–even fucose residues conjugated with antibodies can be transferred onto the cell surface (42) - it is also possible to use this enzyme to incorporate other functional molecules, e.g., cytokines and growth factors, onto NK cells to further boost their *in vivo* properties. Recently, PDGFD-PDGFRβ signal was proven to promote IL-15 mediated NK cell survival (43). We observed no significant change of PDGFRB-PDGFD interactions in scRNA-seq analysis by Cellphone DB, which was consistent with our results that fucosylation would not affect the apoptosis and proliferation of NK cells in GVT model. In conclusion, one of the key factors that govern the success of NK cell-based cancer immunotherapy is achieving efficient early trafficking of an adequate number of activated NK cells into the tumor microenvironment for mounting an effective immune response. *Ex vivo* cell-surface fucosylation certainly serves as a promising strategy to realize this goal.

## Data Availability Statement

The scRNA-seq data presented in the study are deposited in the GEO database (NCBI) repository, accession number GSE182434.

## Ethics Statement

The animal study was reviewed and approved by the Animal Care and Use Committee of Soochow University.

## Author Contributions

PW, HG, JC, and DW designed the study; XT, YR, JF, and YW performed the research; JZ, YD, FL, MY, XiyW, CL, XL, LL, XiaW, LG, and YX contributed to the experiments; XT and HG analyzed the data; XT, HG, JC, and DW wrote the manuscript. PW revised the manuscript critically for important intellectual content.

## Funding

This work was supported by the National Natural Science Foundation of China (No. 81730003, 81700173, 81974001, 81900180, 81974001 and 82170222), National Science and Technology Major Project (2017ZX09304021), National Key R&D Program of China (2019YFC0840604 and 2017YFA0104502), Key R&D Program of Jiangsu Province (BE2019798), Priority Academic Program Development of Jiangsu Higher Education Institutions (PAPD), Jiangsu Medical Outstanding Talents Project (JCRCA2016002), Jiangsu Provincial Key Medical Center (YXZXA2016002), the Jiangsu “333” Talent Project (BRA2015497), the Jiangsu Social Development Program (BE2018651), the Jiangsu Summit Six Top Talent Person project, Jiangsu Medical Junior Talent Person award (QNRC2016707), the Applied Basic Research Programs of Suzhou City (SYS2018027), Suzhou Science and Technology Program Project (SLT201911), the Jiangsu Natural Science Foundation (BK20211070), the Key Disease Program of Suzhou (LCZX202101), China Postdoctoral Science Foundation (2019M661938), Jiangsu Planned Projects for Postdoctoral Research Funds (2019K098), The Natural Science Foundation of Jiangsu Higher Education Institutions of China (20KJD320001), and Translational Research Grant of NCRCH (2021ZKQC01). PW is supported by the NIH (R35GM139643).

## Conflict of Interest

The authors declare that the research was conducted in the absence of any commercial or financial relationships that could be construed as a potential conflict of interest.

## Publisher’s Note

All claims expressed in this article are solely those of the authors and do not necessarily represent those of their affiliated organizations, or those of the publisher, the editors and the reviewers. Any product that may be evaluated in this article, or claim that may be made by its manufacturer, is not guaranteed or endorsed by the publisher.
